# Acute Mastoiditis Associated with *Pseudomonas Aeruginosa* in the Pediatric Population of the Umbria Region, Italy

**DOI:** 10.3390/pathogens8040180

**Published:** 2019-10-09

**Authors:** Guido Camanni, Sonia Bianchini, Cosimo Neglia, Antonella Mencacci, Laura Baldoni, Alessandra Pacitto, Maurizio Stefanelli, Elisabetta Cortis, Susanna Esposito

**Affiliations:** 1 Paediatric Unit, Ospedale San Giovanni Battista, 06034 Foligno, Italy; guido.camanni@uslumbria2.it (G.C.); maurizio.stefanelli@uslumbria2.it (M.S.); elisabetta.cortis@aslroma2.it (E.C.); 2 Paediatric Clinic, Department of Surgical and Biomedical Sciences, Università degli Studi di Perugia, 06123 Perugia, Italy; Bianchini.sonia@fastwebnet.com (S.B.); negliamino@gmail.com (C.N.); Alessandra-pacitto@libero.it (A.P.); 3 Microbiology Unit, Department of Medicine, Università degli Studi di Perugia, 06123 Perugia, Italy; Antonella.mencacci@unipg.it; 4 Microbiology Unit, Ospedale San Giovanni Battista, 06034 Foligno, Italy; laura.baldoni@uslumbria2.it; 5 Pietro Barilla Children’s Hospital, Department of Medicine and Surgery, University of Parma, 43126 Parma, Italy

**Keywords:** acute mastoiditis, acute otitis media, *Pseudomonas aeruginosa*, spontaneous tympanic membrane perforation

## Abstract

Acute mastoiditis (AM) is the most common complication of acute otitis media (AOM) and is one of the most severe acute bacterial diseases in infants and children. In some geographic areas, the incidence of AM is increasing, and the causative role of some bacterial pathogens could be greater than previously thought. In this paper, the results of a study that evaluated the epidemiology and microbial etiology of paediatric AM in Umbria, which is a region of central Italy, are reported. This is a retrospective study of patients aged 0–14 years with AM admitted to the pediatric wards of the hospitals of Umbria, Italy, between June 1 and September 30 in four consecutive years (2015–2018). A total of 108 children were enrolled. The prevalence of AM in males during the four years of analysis was significantly higher than that in females at 63% (95% confidence intervals [CI]: 0.54–0.72). The most frequently affected age groups were 5–9 years (45.4%) and 10–14 years (31.5%), with statistically significant differences in comparison with children aged <1 year (5.6%, 95% CI: 0.01–0.10) and 1–4 years (17.6%, 95% CI: 0.10–0.25). In most cases (64, 59.3%), AM was associated with spontaneous tympanic membrane perforation (STP). The culture of the middle ear fluid revealed the presence of *Pseudomonas aeruginosa* in 56 cases (51.6%). The mean incidence rates of pediatric AM in Umbria during the study increased significantly with time, as it was 18.18/100,000 children/year in 2015–2016 and 29.24/100,000 children/year in 2017–2018 (CI difference: +2.5 – +19.9, p < 0.05). The incidence rates of *Pseudomonas aeruginosa* detection in pediatric AM associated with STP significantly increased with time. The incidence was 6.06/100,000 children/year in 2015–2016 and 18.61/100,000 children/year in 2017–2018 (CI difference: +6.1 – +19.0, p < 0.001). This study demonstrated the high and increasing incidence of AM in the Umbria region during the summer months and the frequent detection of *P. aeruginosa* as an etiologic agent of the disease in the presence of STP. Confirmation of these results with a larger study population, in different settings, and throughout the whole year is needed to define the first-line approach of AM with STP in pediatrics.

## 1. Introduction

Acute mastoiditis (AM) is the most common complication of acute otitis media (AOM) and is one of the most severe acute bacterial diseases of infants and children [1,2]. Prompt diagnosis and effective therapy for AM are mandatory to avoid the risk of life-threatening acute complications and long-term or permanent neurological problems. After it was shown that more than 70% of AOM cases are of bacterial origin and that it is clinically impossible to distinguish viral cases from bacterial cases, it was accepted that the majority of AOM examples require antibiotic treatment. As a result, the incidence and clinical relevance of AM significantly declined. Starting at values up to 20%, the percentage of progression from AOM to AM declined to 0.4%, while the need for hospitalization was halved [3]. This explains why the focus on AM was reduced for many years.

Recently, a number of epidemiological and microbiological reports have again focused on AM. There are two main reasons for this phenomenon. The report shows that, at least in some geographic areas, the incidence of AM may be slightly but significantly increasing, and that the causative role of some bacterial pathogens could be greater than previously thought. Evidence indicated that the increase in the incidence of AM was mainly ascribed to the reduction of antibiotic prescriptions for AOM treatment, as suggested by the watchful waiting practice included in several official guidelines [4,5]. Moreover, the emergence of pathogens resistant to commonly used antibiotics and the widespread use of pneumococcal conjugate vaccines (PCVs) were thought to have played a role [6,7].

Regarding causative bacteria, contrary to what was expected after PCV introduction, the incidence of *Streptococcus pneumoniae* cases was not reduced. Moreover, it was reported that *Pseudomonas aeruginosa*, which is generally considered a causative agent of chronic mastoiditis or AM in children with a history of recurrent AOM, could also be frequently found in AM cases in otherwise healthy children [8,9,10]. Confirmation of these findings seems essential to assure the most effective antibiotic therapy for children with AM. In this paper, the results of a study that evaluated the epidemiology and microbial etiology of pediatric AM in Umbria, which is a region in central Italy, are reported.

## 2. Patients and Methods

This is a retrospective study of patients aged 0–14 years with AM admitted to the pediatric wards of the hospitals of Umbria, which is a region of central Italy, during the period from June 1 – September 30 for four consecutive years (2015–2018). The main aims of the study were to evaluate the incidence rates of AM in the pediatric population and the etiology of the disease. During the study years, approximately 880,000 people lived in the Umbria Region, among whom 112,000 were children aged 0–14 years. All hospitals in Umbria are general hospitals and not children’s hospitals. The ward in Perugia with 21 pediatric beds had more than 10,000 visits in emergency rooms per year, which represented the Hub. Spokes were the ward in Foligno (15 pediatric beds and more than 5,000 visits in emergency rooms) and the ward in Terni (12 pediatric beds and more than 7,000 visits in emergency rooms). Since no other cases of pediatric AM were admitted to Umbria hospitals during the study periods, the incidence rates could be precisely calculated. We decided to study only the summer period because, between June and September in 2015, we observed several cases of AM with spontaneous tympanic membrane perforation (STP) and it was possible to evaluate AM etiology, differently from cases reported during the rest of the year in Italy. The protocol was approved by the Ethics Committee of Umbria (PED-2019-05). Written informed consent was obtained from the parents/legal guardian of each enrolled child and from every enrolled subject aged ≥8 years.

The patient records of cases that fulfilled the criteria for AM were selected. AM was diagnosed based on clinical criteria, including a history of a recent episode of AOM, defined according to the American Academy of Pediatrics and Italian guidelines [11,12], and the presence of at least two of the following retro-auricular signs and symptoms of infection: pain, redness, swelling, fluctuation, and antero-inferior protrusion of the pinna. Patients with primary or secondary immunodeficiency, cranio-facial malformations, concurrent or previous cholesteatoma, cochlear implants in the affected ear, or accidental findings of AM on radiological exams performed for other reasons were excluded.

The analyzed data included age, sex, medical history, laboratory tests, bacterial cultures, antibiotic and surgical treatment, duration of hospital stay, complications, and sequelae. Since, in all the pediatric wards of Umbria hospitals, pediatric AM was managed according to the algorithm proposed by Psarommatis et al. [13], the data collected in the different centers were pooled. In particular, myringotomy was performed only in cases of very severe AM that did not respond to standard antibiotic therapy within 48–72 hours from hospital admission. Moreover, in all cases of spontaneous tympanic membrane perforation (STP), middle ear fluid (MEF) was obtained within 12 hours of STP after the bulk of the otorrhoea fluid had been removed and the ear canal had been cleansed with a dry cotton swab [14]. Under direct otoscopic visualization, the remaining MEF was collected from very near the perforation using an extra-thin flexible wire swab. Samples were immediately sent to the central laboratory of each hospital for culturing. Diagnostic imaging, in the form of computed tomography scans (CT) and/or magnetic resonance imaging (MRI), were performed only for patients with suspected intracranial complications.

In the analysis of collected data, two distinct time periods were considered: 2015–2016 and 2017–2018. The incidence of AM was calculated by dividing the number of hospital discharges for the population at risk, which is represented by the population aged 0–14 years residing in the Umbria region as of 1 January of each year of analysis. Data were extracted from the national Demo-Italian National Institute of Statistics (ISTAT) database. Statistical analysis was performed using Stata Statistical Software: Release 11 (College Station, TX: StataCorp LP). Confidence intervals at the 95% level (95% CI) were calculated to evaluate the population proportion of sex, age classes, and positivity for *Pseudomonas aeruginosa* and ear swab tests. Quantitative variables such as age, hemato-chemistry, duration of intravenous days, and duration of hospitalization were compared between the two groups (positive and negative for *P. aeruginosa*) by using the Student’s t-test. Dichotomous variables were compared using the chi-square test. The proportions of each sex, age class, previous antibiotic treatment, prescription of topical antibiotic, and type of prescribed systemic antibiotic group were compared with the Stata Proportion test.

## 3. Results

A total of 108 children were enrolled. Table 1 shows the total hospital admissions for AM per study year with the general characteristics of the study population. The prevalence of AM in males during the four years of analysis was significantly higher than in females: 63% (95% CI: 0.54–0.72). The most frequently affected age groups were 5–9 years (45.4%) and 10–14 years (31.5%), with statistically significant differences in comparison with <1 year (5.6%, 95% CI: 0.01–0.10) and 1–4 year groups (17.6%, 95% CI: 0.10–0.25). In most cases (64, 59.3%), AM was associated with STP. The bacterial etiology of AM could be established only in these cases, as no myringotomies or mastoidectomies were performed. MEF cultures revealed the presence of *P. aeruginosa* in 56 cases (51.6%). *P. aeruginosa* alone was cultured in 49 (45.4%) children. In seven children (6.5%), it was associated with other AOM pathogens. In eight (7.4%) children, the cultures were negative. None of the enrolled patients had a history of recurrent AOM.

Moreover, the mean incidence rates of pediatric AM in Umbria during the study period significantly increased with time, from 18.18/100,000 children/year in 2015–2016 to 29.24/100,000 children/year in 2017–2018 (CI difference: +2.5 – +19.9, p < 0.05). The incidence rates of *P. aeruginosa* detection in pediatric AM associated with STP increased significantly with time, from 6.06/100,000 children/year in 2015/2016 to 18.61/100,000 children/year in 2017–2018 (CI difference: +6.1 – +19.0, p < 0.001). All children were given intravenous antibiotic treatment.

Table 2 shows the clinical characteristics of enrolled patients stratified by *P. aeruginosa* positivity in MEF. The mean age was significantly higher in the *P. aeruginosa*-positive patients, whereas the sex distribution was similar in the two groups. WBC counts and CRP levels were significantly lower in the *P. aeruginosa*-positive patients than in the negative patients. More than half of the study population was previously treated with oral antibiotics in the 48 h before hospital admission. No significant differences were observed in topical antibiotic prescriptions, whereas the duration of intravenous treatment and hospitalization were significantly longer in the *P. aeruginosa*-positive group than in the negative group. A minority of patients in both groups underwent CT or MRI, and no surgical procedures were required.

All *P. aeruginosa* strains were susceptible to gentamicin, ceftazidime, and piperacillin-tazobactam. In addition, 55 (98.2%) strains were susceptible to ciprofloxacin and 52 (92.8%) strains were susceptible to imipenem and meropenem. Lastly, 51 (91.1%) strains were susceptible to amikacin. Resistance to two antimicrobial groups was not common and was observed in 2 (3.6%) strains of the positive isolates.

There was no resistance to three or more antimicrobial groups.

## 4. Discussion

This study shows that, in Umbria, which is a region of central Italy, the incidence of pediatric AM during summer months is particularly high and progressively increasing, and the frequent detection of *P. aeruginosa* as an etiologic agent of the disease in the presence of STP. Increases in the incidence of pediatric AM have already been reported in several countries, including Italy. However, data collected in Umbria indicate a significantly higher incidence, and this increase seems strictly related to the increase in the incidence of *P. aeruginosa* cases.

The role of *P. aeruginosa* in the pathogenesis of AM with STP has been considered marginal for several years, but a number of recent reports indicate that *P. aeruginosa* can play a role in a greater number of cases than previously thought. This is clearly exemplified by the studies of Butbul-Aviel et al. [15] and Laulajainen-Hongisto et al. [8], who found *P. aeruginosa* in the MEF of 25% and 11% of children with AM, respectively. In these studies, the present study found that *P. aeruginosa* cases were more common among older children and had a relatively benign course, as signs and symptoms of disease receded in a few days under appropriate antibiotic therapy, and mastoidectomy was not required for any of the children.

The relevance of *P. aeruginosa* as a cause of AM confirms what has been already reported by Luntz et al., who clearly showed that the distribution of causative bacteria in AM can differ from the distribution in AOM [16]. This raises the problem of whether the empiric treatment of AM must be changed. Usually, antibiotics are prescribed with the assumption that *S. pneumoniae* is the most likely pathogen. However, the drugs that are usually prescribed are ineffective against *P. aeruginosa,* and late treatment can lead to a negative evolution. Further studies concerning the etiology of pediatric AM and the role of *P. aeruginosa* in cases with and without STP are needed.

This study has some limitations. First, the analysis considered a limited period of the year. Second, the examination of the etiology of the disease was limited to cases with STP. However, the study represents an important real-life experience because the high number of AM cases with STP and *P. aeruginosa* positivity in MEF during the summer months alerts pediatricians working in Umbria hospitals for the need to perform a retrospective evaluation. Moreover, the incidence was precisely calculated, and the resulting data indicate that, despite a progressive increase, the incidences were quite similar in the different years, which suggests reliability.

## 5. Conclusions

This study demonstrated the high and increasing incidence of AM in the Umbria region during the summer months and the frequent detection of *P. aeruginosa* as the etiologic agent of the disease in the presence of STP. Confirmation of these results with a larger study population, in different settings, and during the whole year is needed to define the first-line approach for AM in pediatric services.

## Figures and Tables

**Table 1:** General characteristics of patients enrolled with a diagnosis of acute mastoiditis (AM) during the years 2015-2018.

	2015	2016	2017	2018	Total	95% CI	P-Value
**Hospital admissions**	28	14	38	28	108	-	-
**Male, N (%)**	15 (53.6)	11 (78.6)	25 (65.8)	17 (60.7)	68 (63.0)	[0.54–0.72]	**<0.01**
**Age group, N (%)**							
<1	1 (3.6)	0 (0.0)	5 (13.2)	0 (0.0)	6 (5.6)	[0.01–0.10]	
1–4	5 (17.9)	5 (35.7)	6 (15.8)	3 (10.7)	19 (17.6)	[0.10–0.25]	
5–9	16 (57.1)	6 (42.9)	14 (36.9)	13 (46.4)	49 (45.4)	[0.36–0.55]	
10–14	6 (21.4)	3 (21.4)	13 (34.2)	12 (42.9)	34 (31.5)	[0.23–0.40]	
***Pseudomonas aeruginosa* positive, N (%)**	9 (32.1)	5 (35.7)	24 (63.2)	18 (64.3)	56 (51.9)	[0.42–0.61]	0.7
**Ear swab positive** **N (%)**	10 (35.7)	6 (42.9)	25 (65.8)	22 (78.6)	63 (58.3)	[0.49–0.68]	0.08

**Table 2:** Clinical characteristics of all enrolled patients stratified by *Pseudomonas aeruginosa* positivity.

	Negative N = 52	Positive N = 56	P-Value
**Sex**			0.089
Male	37 (71.1)	31 (55.3)	0.418
Female	15 (28.9)	25 (44.7)	0.248
**Age, mean**	6.9 ± 4.2	8.8 ± 3.3	**<0.05**
**Age, years**			**<0.05**
<1	4 (7.7)	2 (3.6)	0.377
1–4	14 (26.9)	5 (8.9)	<0.05
5–9	22 (42.3)	27 (48.2)	0.705
10–14	12 (23.1)	22 (39.3)	0.189
**WBC/mm^3^**	13,034.8 ± 6,023.6	9,795.6 ± 3,966.2	**<0.01**
**CRP, mg/dL**	4.2 ± 3.7	2.9 ± 2.8	**<0.05**
**Antibiotics in the 48 hours before admission, yes**	28 (53.9)	38 (67.9)	0.136
Amoxicillin and clavulanic acid	18 (64.3)	22 (57.9)	0.733
Others	3 (10.7)	4 (10.5)	0.786
Missing data	7 (25.0)	12 (31.6)	0.362
**Topical antibiotics, yes**	24 (46.1)	42 (75.0)	0.128
Ciprofloxacin	15 (28.8)	30 (53.6)	0.092
Tobramycin	2 (3.8)	1 (1.8)	0.291
Netilmicin	0 (0.0)	1 (1.8)	0.337
Not reported	7 (13.5)	10 (17.9)	0.592
**Topical antibiotics, days**	8.2 ± 2.9	8.7 ± 2.6	0.4468
**Systemic antibiotics, yes**	52 (100.00)	56 (100.0)	
Ceftazidime	20 (38.5)	42 (75.0)	**<0.05**
Ceftriaxone	21 (40.4)	10 (17.9)	0.054
Piperacillin	3 (5.8)	3 (4.4)	0.93
Others	3 (5.8)	0 (0.0)	0.786
Missing	5 (9.6)	1 (1.8)	0.093
**Duration of intravenous therapy, days**	6.5 ± 2.6	8.3 ± 3.2	**<0.01**
**Duration of hospitalization, days**	7.3 ± 2.1	8.8 ± 2.9	**<0.01**
**CT/MRI, yes**	5 (9.6)	5 (8.9)	0.911

CT, computed tomography. CRP, C reactive protein. MRI, magnetic resonance imaging. WBC, white blood cells.
